# Fouling Mitigation via Chaotic Advection in a Flat Membrane Module with a Patterned Surface

**DOI:** 10.3390/membranes11100724

**Published:** 2021-09-23

**Authors:** Kyung Tae Kim, Jo Eun Park, Seon Yeop Jung, Tae Gon Kang

**Affiliations:** 1 School of Aerospace and Mechanical Engineering, Korea Aerospace University, Goyang-si 10540, Gyeonggi-do, Korea; ktk1636363@gmail.com (K.T.K.); p.joeun@gmail.com (J.E.P.); 2 Department of Chemical Engineering, Dankook University, Yongin-si 16890, Gyeonggi-do, Korea; seon27@dankook.ac.kr

**Keywords:** flat membrane module, crossflow filtration, fouling mitigation, patterned surface, chaotic advection, numerical simulation

## Abstract

Fouling mitigation using chaotic advection caused by herringbone-shaped grooves in a flat membrane module is numerically investigated. The feed flow is laminar with the Reynolds number (Re) ranging from 50 to 500. In addition, we assume a constant permeate flux on the membrane surface. Typical flow characteristics include two counter-rotating flows and downwelling flows, which are highly influenced by the groove depth at each Re. Poincaré sections are plotted to represent the dynamical systems of the flows and to analyze mixing. The flow systems become globally chaotic as the groove depth increases above a threshold value. Fouling mitigation via chaotic advection is demonstrated using the dimensionless average concentration (${\bar{c}}_{w}^{*}$) on the membrane and its growth rate. When the flow system is chaotic, the growth rate of ${\bar{c}}_{w}^{*}$ drops significantly compared to that predicted from the film theory, demonstrating that chaotic advection is an attractive hydrodynamic technique that mitigates membrane fouling. At each Re, there exists an optimal groove depth minimizing ${\bar{c}}_{w}^{*}$ and the growth rate of ${\bar{c}}_{w}^{*}$. Under the optimum groove geometry, foulants near the membrane are transported back to the bulk flow via the downwelling flows, distributed uniformly in the entire channel via chaotic advection.

## 1. Introduction

The filtration of foulants such as molecules or fine particles from a feed fluid using membranes is used in many fields, including environmental, chemical, biological, pharmaceutical, and food industries [1,2]. Recently, membrane filtration has also been used as a carbon capture technology, i.e., the processes of capturing carbon dioxide (CO_2_) and reducing the emission of CO_2_ [3]. Depending on the relative direction between the feed flow and the permeate flow in a membrane filtration process, filtration is classified into dead-end filtration and crossflow filtration. The feed flow is perpendicular to the membrane surface in a dead-end filtration system, while the feed flow is tangential to the flat or tubular membrane in a crossflow filtration (CFF) system. The permeate flux is produced by the transmembrane pressure difference across the membrane. The accumulation of foulants on a membrane surface (membrane fouling) increases the resistance to filtration, leading to a decline in the permeate flux and lifespan of the membrane. Membrane fouling is caused by particle intrusion into membrane pores, narrowing or blocking flow passages through the pores, or by an adsorption of molecules on the membrane surface, forming a boundary layer with higher concentrations than those in the bulk flow [4,5,6,7,8].

Though fouling is an inevitable drawback of membrane filtration, there are several ways to effectively alleviate membrane fouling and maintain filtration performance for as long as possible. One approach is to modify the membranes to give them anti-fouling properties, but this requires additional engineering processes in the manufacturing stage [9,10,11]. The other approach to mitigate membrane fouling is to apply hydrodynamic techniques that utilize vortical flows, pulsatile flows, turbulent flows, or chaotic advection in laminar flows, enabling the back-transport of foulants from the region near the membrane surface to the bulk flow [6]. In crossflow filtration, especially, specially designed inserts or channel geometry can induce such flows. Typical examples that fall into this category are a curved channel creating Dean vortices [12], a tubular membrane with a helical baffle [13], a tubular membrane with mixing elements [14,15,16,17], and a microchannel with patterned surfaces inducing chaotic advection [18]. The present study employs a crossflow filtration module that consists of a flat membrane and a patterned non-permeable solid surface with herringbone-shaped grooves, which stirs the feed fluid via chaotic advection, thereby suppressing fouling and concentration polarization in the crossflow filtration module. For more details on chaotic advection, we refer to three review papers [19,20,21]. 

Patterned solid surfaces are adopted in a variety of applications to enhance mixing, heat transfer, or filtration performances. In passive mixing, for example, a properly designed surface pattern can induce lateral flows in addition to a primary axial flow, repeating periodically and ultimately leading to efficient mixing in the low Reynolds number flow, where mixing is governed by molecular diffusion rather than convective mass transport. The staggered herringbone mixer (SHM) [22], the barrier-embedded micromixer (BEM) [23,24], and the groove-embedded partitioned pipe mixer (GPPM) [25] are representative examples utilizing patterned surfaces to achieve enhanced mixing at low Reynolds flow. In electronic cooling, micro-structured heat sinks are used to achieve better convective heat transfer coefficients, resulting in the heat transfer enhancement [26]. The additional flow created by the microstructured surface plays a key role in improving cooling performance. The detailed fluid flow over a patterned surface varies with surface morphology. 

As far as patterned membranes are concerned, various patterns have been employed to achieve enhanced anti-fouling properties. In wastewater treatment, a prism-shaped or pyramid-shaped morphology was introduced on a membrane surface to mitigate fouling [27]. Jung and his co-workers conducted numerical and experimental studies of the particle deposition on a patterned membrane surface [28,29,30]. Using Brownian dynamics simulation, they reproduced particle deposition observed in the experiment. Bixler et. al. developed and characterized bio-inspired anti-fouling surfaces mimicking rice leaves and butterfly wings, combining anti-fouling and self-cleaning effects [31]. Choi et. al. fabricated sharkskin-mimetic reverse osmosis (RO) membranes and characterized the bio-fouling properties affected by the patterned membranes [32]. Recently, they also conducted numerical studies on the flow and the colloidal fouling behavior on the sharkskin-mimetic surfaces [33,34]. It was found that to obtain optimum process conditions that minimized fouling on the patterned membrane, both the length scale of the surface pattern and that of the foulants had to be considered together. The above-mentioned studies on patterned membranes have revealed that the ratio between the pattern size and the length scale of other parameters such as particle size and channel height has a significant influence on fouling mitigation relying on the hydrodynamics of a feed fluid due to a specific membrane pattern. Computational fluid dynamics (CFD) simulations can be used to elucidate the influence of surface features on the hydrodynamics and concentration polarization near the membrane surfaces [35,36]. 

In this study, we attempt to numerically characterize the fouling mitigation via chaotic advection in a crossflow filtration module consisting of a flat membrane and solid surfaces with herringbone-shaped grooves and then demonstrate the feasibility of chaotic advection as a hydrodynamic technique mitigating membrane fouling. In a previous study [18], we conducted numerical simulations to optimize the design parameters of a microfluidic filtration module incorporating the staggered herringbone mixer (SHM) working in the creeping flow regime. Using the Taguchi method and computational fluid dynamics (CFD) software, an optimal set of design parameters minimizing the growth rate of the wall concentration was identified. In addition, it turned out that the groove depth was the most influential factor to effectively suppress the concentration growth using chaotic advection. The previous study successfully demonstrated that chaotic advection could be a promising option to mitigate fouling in crossflow filtration. As an extension of the previous study, we now attempt to elucidate the effect of the groove depth on fouling mitigation in the flat membrane module, while extending the operating conditions to non-creeping flows where inertia plays an important role in the flow and mass transfer. Enhanced mixing via chaotic advection of the feed fluid is expected to be a key mechanism mitigating fouling and concentration polarization. This is because chaotic advection leads to chaotic trajectories of fluid particles and foulants, thereby uniformly dispersing the foulants in the channel. In this regard, a systematic numerical investigation is carried out to understand the flow characteristics and the mass transport in the crossflow filtration module with a flat membrane and a patterned non-permeable solid surface. 

The present paper is organized as follows. First, the channel geometry is introduced, followed by the governing equations and the boundary conditions required to solve the flow and mass transport problems. A proper number of elements and the minimum element size, showing convergence with mesh refinement, are determined. Next, the flow characteristics within a periodic domain, which are affected by the dimensionless groove depth and the Reynolds number, are presented using the cross-sectional streaklines, the downwelling velocity magnitude, and Poincaré sections. As for the mass transfer in the filtration module, the focus will be on the effects of two parameters (i.e., the dimensionless groove depth and the Reynolds number of the bulk flow) on the concentration distribution on the membrane surface and the growth rate of the wall concentration along the down-channel direction. The correlation between the flow characteristics (that can be estimated by the cross-sectional flows and Poincaré sections) and the membrane fouling (characterized by the dimensionless concentration and its growth rate) will be discussed in detail. Finally, the pressure loss in the filtration module is also identified to assess the energy consumption influenced by the groove depth and fluid inertia.

## 2. Problem Definition

### 2.1. Channel Geometry

**Figure 1:**
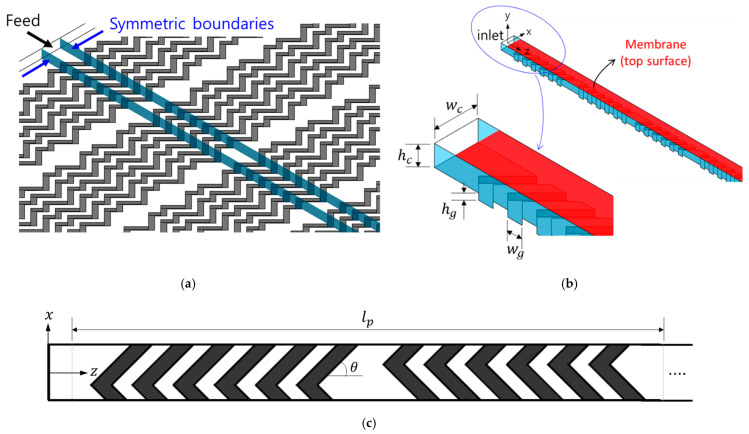
The schematic representation of the flat membrane module consisting of a patterned solid surface (bottom surface) and a flat membrane (top surface). (**a**) The non-permeable patterned surface with staggered herringbone-shaped grooves; (**b**) The channel geometry with symmetry in the lateral direction (x‒direction) used in numerical simulations and an enlarged image around the inlet. In this figure, the top surface is the membrane (the area shaded in red); (**c**) Top view of the periodic unit of the channel, where groove regions are shaded. The channel is geometrically periodic in the z‒direction with the period $l_{p}$. In each half cycle, there are six grooves. Here, $h_{c}$, $h_{g}$, $w_{c}$, $w_{g}$, and θ are the channel height, the groove depth, the channel width, the groove width, and the groove angle, respectively. The groove angle is fixed to θ=45°.

The flat membrane module consisting of a non-permeable patterned solid surface and a flat membrane is schematically depicted in Figure 1. A herringbone-shaped pattern is fabricated at the non-permeable bottom surface, while the top surface of the channel is a flat membrane (Figure 1a). The pattern is symmetric in the lateral direction (x‒direction) and periodic in the feed flow direction (z‒direction). Figure 1b depicts the entrance region of the computational domain. The length of the periodic channel geometry is denoted by $l_{p}$ (Figure 1c). In this study, a filtration module consisting of 10 periodic units is used as a computational domain in characterizing the foulant concentration on the membrane and its development in the z‒direction. In each half period of the channel, six grooves pattern the bottom surface and are placed with the groove angle (θ) being 45° with respect to the channel axial direction. The channel height, the groove depth, the channel width, and the groove width are denoted by $h_{c}$, $h_{g}$, $w_{c}$, and $w_{g}$, respectively. The channel aspect ratio, $h_{c}/w_{c}$, is fixed to be 10/21, and the grove width $w_{g}$ is $0.35\;w_{c}$. As depicted in Figure 1c, the apex of a groove is located at $1/3\;w_{c}$ from the left sidewall in the first half period and at $1/3\;w_{c}$ from the right sidewall in the next half period, forming a staggered herringbone pattern, which is the same as the original SHM design [22]. 

In a previous study [18] on the optimization of a microfluidic filtration module incorporated with the SHM, it turned out that the groove depth was the most influential geometrical variable that affected fouling. Additionally, in a parameter study on mixing in the SHM [37,38], the depth ratio of the groove was found to be the most influential factor. Though other design variables such as the groove angle, groove asymmetry, number of grooves per period, and groove width may influence the flow and mixing, their influences are not as significant as that of the groove depth. In this regard, we chose the dimensionless groove depth $h_{g}^{*}$ ($=h_{g}/h_{c}$) as the only geometrical variable, and its influence on the flow and mass transfer near the membrane surface will be studied in this paper.

### 2.2. Governing Equations and Boundary Conditions

In the flat membrane module, the feed fluid is introduced through the inlet in the z‒direction. To obtain the concentration evolution in the down-channel direction, the flow and mass transfer problems should be solved. The fluid is assumed to be incompressible and Newtonian, neglecting gravity. In this study, we solve the steady Navier–Stokes equation with the incompressibility constraint and the convection–diffusion equation for the mass transfer in a decoupled manner. The continuity and the Navier–Stokes equations are given by

(1) ∇·u=0

(2) $$\rho \left(u\cdot \nabla u\right)=-\nabla p+\mu \nabla^{2}u$$

where u is the velocity, ρ the density, p the pressure, and μ the viscosity. Since we use the Cartesian coordinate system, the velocity vector is represented by u=(u,v,w)**.** At the inlet ($\Gamma_{i}$), a uniform normal velocity ($\bar{u})$ is imposed, while at the outlet ($\Gamma_{o}$), a constant static pressure (in this study, p=0) is specified. At the non-permeable patterned surface ($\Gamma_{s}$), the velocity is zero, i.e., u=0 on $\Gamma_{s}$. On the membrane surface ($\Gamma_{w}$), a constant permeate flux is imposed, i.e., $u\cdot n=u_{p}$, where n is the unit outward normal vector at $\Gamma_{w}$ and $u_{p}$ the constant permeate velocity on the membrane surface. The Reynolds number (Re) of the bulk flow is defined as Re=ρu¯hc/μ, where the uniform inlet velocity $\bar{u}$ is used as the characteristic velocity, and Re ranges from 50 to 500. In addition, we define another Reynolds number, called the wall Reynolds number (Rew), defined by Rew=ρuphc/μ, employing $u_{p}$ as the characteristic velocity. As for the permeate velocity, a constant $u_{p}$ is used, regardless of the inlet velocity, such that Rew=0.01, corresponding to a constant flux mode of operation.

The concentration of foulants is obtained by solving the steady convection–diffusion equation, given by

(3) ∇·(D∇c)−u·∇c=0,

where D is the foulant diffusivity and c the concentration. Equation (3) represents the mass transport driven by the convection and diffusion of the foulants, neglecting complex transport phenomena such as shear-induced migration, frictional dynamics on the membrane surface, diffusiophoresis, adhesion on surfaces, etc. As a boundary condition at the inlet, a constant concentration ($c_{0}$) is imposed, assuming uniformly distributed foulants in the feed solution. At the outlet, the diffusive flux of the foulants is set to zero, i.e., n·D∇c=0 on $\Gamma_{o}$. The total mass flux of foulants is zero at the membrane surface, i.e., n·(−D∇c+cu)=0 on $\Gamma_{w}$, implying a complete rejection of the foulants by the membrane. The zero-flux condition is imposed on the non-permeable boundary ($\Gamma_{s}$) as well. The Péclet number (Pe), representing the ratio of the convective transport to the diffusive transport, is defined by $\mathrm{Pe}=\bar{u}h_{c}/D$, ranging from $5\times 10^{5}$ to $5\times 10^{6}$ depending on the magnitude of the inlet velocity. Therefore, the convective mass transport governs the foulant distribution within the membrane module. With the permeate flux being fixed, the concentration on the membrane surface reflects fouling. Throughout the paper, we present the results using dimensionless quantities: velocity components are made dimensionless with $\bar{u}$, the concentration with $c_{0}$, the groove depth with $h_{c}$, and the channel length with $l_{p}$. 

### 2.3. Simulation Details

In numerical simulations, commercial CFD software (ANSYS-CFX 18.1, ANSYS Inc., Canonsburg, PA, USA) is used to solve the flow and mass transport problems in the flat membrane module. The flow and mass transport problems are solved in a decoupled manner. A higher-order scheme is employed to prevent numerical instabilities caused by the convection terms in Equations (2) and (3). The computational domain is discretized with hexahedral elements. The proper number of elements is determined by checking the convergence of solutions using the four meshes, M1, M2, M3, and M4, with a different number of elements (see Table 1). The details on the mesh convergence check will be introduced in the following section. In this study, a workstation with a 10-core CPU (Intel^®^ Xeon^®^ Silver 4210R 2.4 GHz) and 98 GB memory is used, and all computations are carried out using parallel computing with 10 processors. 

## 3. Results and Discussion

### 3.1. Convergence with Mesh Refinement

**Figure 2:**
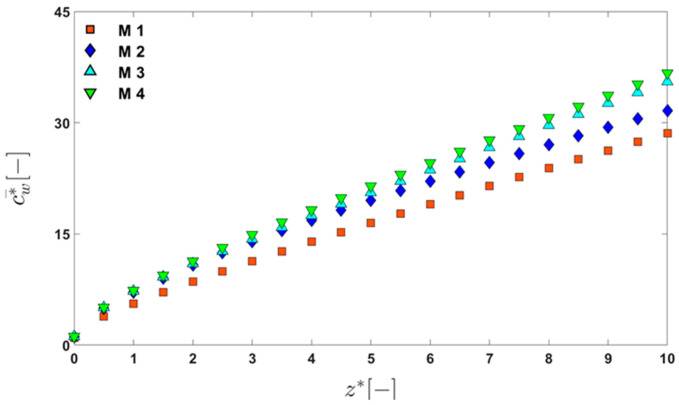
The change in the dimensionless average wall concentration (${\bar{c}}_{w}^{*}$) in the dimensionless z‒direction ($z^{*}$) showing convergence with mesh refinement. Basic information on the four meshes used to check mesh convergence can be found in Table 1.

First, the convergence of the solution with the mesh refinement is verified using a computational domain consisting of 10 periodic units with $h_{g}^{*}=0.05$ and Re=100. The four meshes (M1, M2, M3, and M4) are used to check mesh convergence and to determine a proper number of elements that will be used in simulations. Since the concentration is the most sensitive to the element size in the convection–dominant mass transfer problem, we choose the concentration on the membrane surface ($c_{w}$) to check convergence with mesh refinement. Figure 2 shows the dimensionless line-averaged wall concentration (${\bar{c}}_{w}^{*}$) on the membrane surface, which is defined by

(4) $${\bar{c}}_{w}^{*}=\int_{L}^{}\frac{c_{w}^{*}\;dx}{w_{c}},$$

as a function of the dimensionless z‒coordinate, $z^{*}=z/l_{p}$, where $c_{w}^{*}$ is the dimensionless wall concentration defined by $c_{w}^{*}=c_{w}/c_{0}$. In Equation (4), $c_{w}^{*}$ is integrated along a line segment (L) on the membrane surface, directed in the x‒direction at axial position z. As observed in Figure 2, the dimensionless average wall concentration shows convergence with the mesh refinement. Considering the results of the convergence test, we choose mesh M4 as the reference mesh, from which the number of elements and the element size used in all of the computations are determined. 

### 3.2. Flow Characteristics

Hydrodynamic techniques alleviating fouling in membrane filtration rely on a particular flow occurring in a filtration device [5]. As such, it is crucial to understand the in-depth flow characteristics in the specific channel geometry and the flow conditions of the filtration module. As far as the herringbone pattern in a duct flow is concerned, it has been demonstrated that two asymmetric counter-rotating flows are created by the herringbone-shaped grooves fabricated in a rectangular microchannel working in the creeping flow (Re≪1) [22,37,38,39,40,41,42]. When the two flow portraits are projected onto a single plane normal to the z‒axis, the cross-sectional flow patterns from the two flows intersect each other, satisfying the necessary condition for chaotic advection in a three-dimensional geometrically periodic flow [20,21,43]. In the case of the flat membrane module (Figure 1), unlike the two side walls of the microchannel, the two side surfaces of the computational domain (depicted in Figure 1b) are not rigid walls but open boundaries, where a symmetric boundary condition is applied. Thus, the fluid is allowed to slip along the side boundaries. Contrary to the microchannel flow, in addition, the fluid flow is laminar with the Reynolds number ranging from 50 to 500, where inertia plays an important role in the flow and the convective mass transfer. 

**Figure 3:**
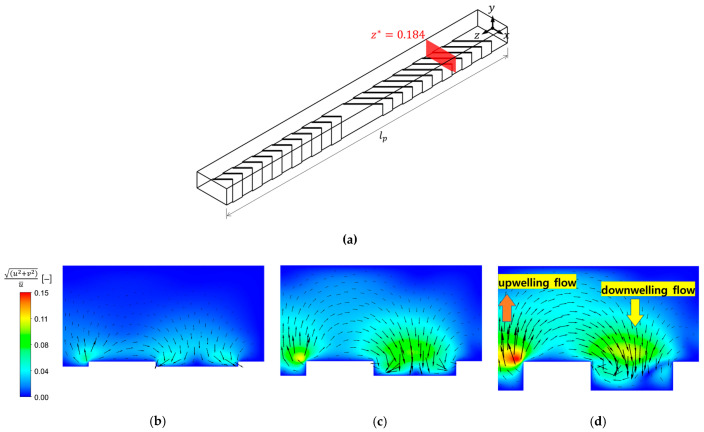
The cross-sectional velocity vectors and the magnitude of the cross-sectional velocity at the cross section located at $z^{*}=0.184\;$(shown in (**a**)), scaled by the average inlet velocity, $\sqrt{\left(u^{2}+v^{2}\right)}/\bar{u}$, representing the strength of the cross-sectional flow when Re=100: (**a**) the location of the cross section where the velocity vectors and velocity contours are plotted for the three groove depths; (**b**) $h_{g}^{*}=0.05$; (**c**) $h_{g}^{*}=0.15$; (**d**) $h_{g}^{*}=0.3$. The maximum values at the three values of $h_{g}^{*}$, 0.05, 0.15, and 0.3, are 0.059, 0.115, and 0.152, respectively. The arrows in each contour plot represent the velocity vector projected onto the cross section (viewed from the outlet).

To understand the flow characteristics due to the patterned surface, a periodic channel repeating in the z‒direction is chosen (Figure 3a), and the velocity field in the periodic domain is obtained by solving Equations (1) and (2). In this Section only, we use no-slip and non-penetration conditions for the upper surface $\Gamma_{w}$ (corresponding to the membrane), focusing on the flow affected by the grooves and inertia. Additionally, a periodic boundary condition is applied at the inlet and outlet to obtain a periodic flow field, ensuring the geometric periodicity of the channel in the z‒direction. A fixed inlet flow rate is imposed in addition to the constraint for the periodic velocity. The typical flows in the flat membrane module with a periodically repeating herringbone-shaped surface pattern are also two counter-rotating flows, which will be significantly influenced by the groove depth and inertia. To demonstrate the flow characteristics affected by the groove depth and inertia, three dimensionless groove depths ($h_{g}^{*}=0.05$, 0.15, and 0.3) are chosen at Re=100, and the velocity fields for the three cases are obtained. The obtained periodic velocity fields for the three grooves depths are used to plot Poincaré sections and to estimate asymptotic mixing behaviors, which will be discussed shortly. 

Figure 3b–d show the cross-sectional velocity vectors and the magnitude of the cross-sectional velocity relative to the average inlet velocity, defined by $\sqrt{\left(u^{2}+v^{2}\right)}/\bar{u}$, at $h_{g}^{*}=0.05$, 0.15, and 0.3, respectively, when Re=100. The cross section is located at an axial position with $z^{*}=0.184$ (shaded section in Figure 3a). The contour plots represent the strength of the in-plane motions of the fluid in the cross section, while the arrows in the three plots represent the cross-sectional velocity vectors viewed from the outlet. At this Reynolds number (Re=100), the strength of the rotational flows increases as the groove depth increases. Near the apex of the groove, one can observe downwelling flows transporting the fluid to the left and the right at the groove region. Along the two side surfaces, on the other hand, upwelling flows occur. The periodically alternating upwelling and downwelling flows induced by the periodic groove pattern result in alternating two asymmetric counter-rotating flows in an overall sense, enabling chaotic advection in the periodic flow.

**Figure 4:**
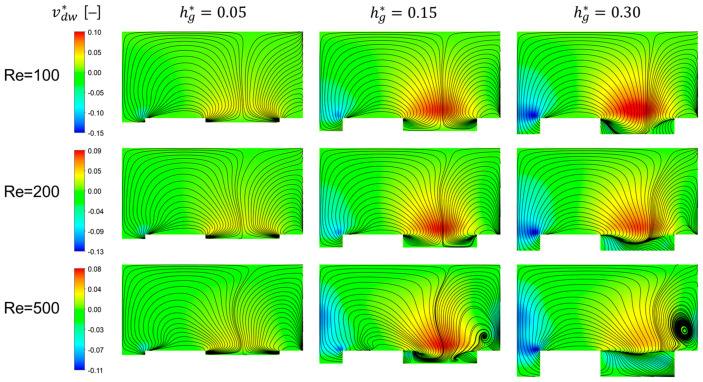
Cross-sectional flows at the cross section located at $z^{*}=0.184$ visualized by streaklines and contours of the downwelling velocity magnitude affected by the dimensionless groove depth ($h_{g}^{*}$) and the Reynolds number (Re). In contour plots, the dimensionless downwelling velocity magnitude, defined by $v_{dw}^{*}=-v/\bar{u}$, is plotted. While positive contours indicate downwelling flows, negative contours indicate upwelling flows. In each cross-sectional plot, the upper edge corresponds to the membrane.

We now wish to examine the downwelling and upwelling flows in more detail since they are closely related to the mitigation of the fouling and concentration polarization in the filtration module using the patterned surface. The dimensionless downwelling velocity magnitude, defined by $v_{dw}^{*}=-v/\bar{u}$, is used to characterize the strength of downwelling motions of the fluid. Figure 4 shows cross-sectional flow patterns visualized by streaklines and contours of the dimensionless downwelling velocity magnitude ($v_{dw}^{*}$) at three Reynolds numbers (Re=100, 200, and 500) by varying the dimensionless groove depth $h_{g}^{*}$. In the cross-sectional images, the upper edge corresponds to the membrane surface. Note that in the contour plots, positive values of $v_{dw}^{*}$ indicate downwelling flows and negative values indicate upwelling flows. 

When Re=100, as the depth of the groove increases, the strength of the downwelling flow increases. At the other two Reynolds numbers, Re=200 and 500, however, the downwelling velocity magnitude at the deepest groove (with $h_{g}^{*}=0.30$) is not the largest. When Re=200, $v_{dw}^{*}$ increases notably as $h_{g}^{*}$ increases from 0.05 to 0.15, but the increase of $v_{dw}^{*}$ becomes saturated as $h_{g}^{*}$ increases from 0.15 to 0.3. When Re=500, interestingly, $v_{dw}^{*}$ decreases significantly as $h_{g}^{*}$ increases from 0.15 to 0.3. In addition, the upwelling velocity magnitude is larger than the downwelling velocity magnitude, which would have a negative influence on fouling mitigation. As the groove depth increases, the separatrix dividing the two rotational flows in a cross section moves towards the left regardless of the Reynolds number. Quantitative analyses of the combined effect of the unique flow characteristics in the flat membrane module (the two counter-rotational flows with downwelling and upwelling flows) on the concentration distribution and fouling mitigation characterized by the growth rate of the wall concentration will be introduced in Section 3.3 and Section 3.4.

The dynamical systems of the periodic flows can be examined by plotting Poincaré sections, which can also be used to analyze mixing behaviors [20]. A flow system is globally chaotic if there are no visible islands in the corresponding Poincaré section obtained using the periodic velocity field, while if a Poincaré section possesses any visible island, then mixing in the island is regular (not chaotic). It is an interesting subject to relate the chaotic behavior of the feed fluid to the fouling mitigation for the flat membrane module. If a flow system is chaotic, fluid elements exhibit strong deformations due to repeated stretching and folding, resulting in an exponential growth of the distance between two neighboring fluid particles [19,20,21]. Once chaotic advection occurs in a filtration module working in a laminar flow, it can uniformly distribute foulants via chaotic mixing, thereby mitigating membrane fouling if properly combined with downwelling flows [16]. The periodic velocity field obtained for a specific set of $h_{g}^{*}$ and Re is used to plot the Poincaré section for the flow system. When tracking tracer particles to plot Poincaré sections, a 5th-order Runge–Kutta method derived by Cash and Karp [44] is used to ensure the accuracy of particle trajectories. 

**Figure 5:**
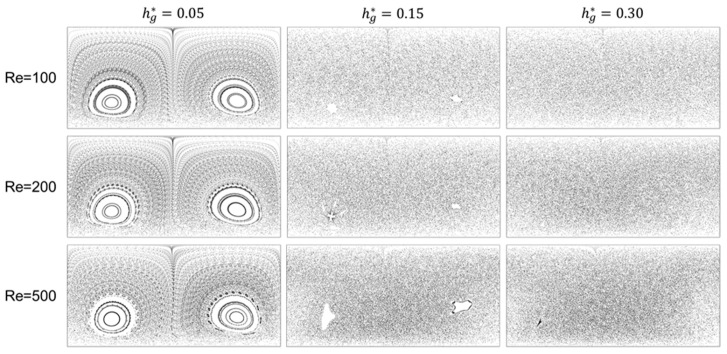
Poincaré sections affected by the dimensionless groove depth ($h_{g}^{*}$) at the three Reynolds numbers, Re=100, 200, and 500.

Figure 5 depicts the Poincare sections for the three groove depths, $h_{g}^{*}=0.05$, 0.15, and 0.3, when Re=100, 200, and 500, demonstrating changes in the dynamical systems. For a fixed groove depth, the detailed structure of the Poincaré section varies with Re, but the change in the overall degree of chaos is negligible with the increase in Re from 100 to 500. In addition, the change in Poincaré sections with the increase of the groove depth is quite similar, regardless of Re: from regular (at $h_{g}^{*}=0.05$) to partially chaotic (at $h_{g}^{*}=0.15$) and to globally chaotic (at $h_{g}^{*}=0.3$). At the lowest groove depth, $h_{g}^{*}=0.05$, two islands surrounded by Kolmogorov–Arnold–Moser (KAM) boundaries [19,20] are clearly observed. Thus, the flow system is regular rather than chaotic. At this groove depth, accordingly, one cannot expect an effective stirring to mitigate fouling. As the groove depth $h_{g}^{*}$ increases from 0.05 to 0.15, the two islands become noticeably smaller, and the flow system is almost chaotic except for the two tiny islands. At the deepest groove with $h_{g}^{*}=0.3$, the two islands disappear completely, and the flow system is thus globally chaotic, enabling the fluid particles and foulants to be uniformly redistributed in the entire channel. At each Re, the flows at the two groove depths, $h_{g}^{*}=0.15$ and 0.3, are expected to significantly reduce membrane fouling and concentration polarization compared to the filtration module with the plain surface (without grooves). Because a Poincaré section reveals the asymptotic behavior of a flow system, the evolution of the concentration (for a filtration module with a finite length) in the feed direction should be evaluated to confirm the effectiveness of a globally (or partially) chaotic flow on fouling mitigation.

### 3.3. Evolution of the Foulant Concentration 

**Figure A1:**
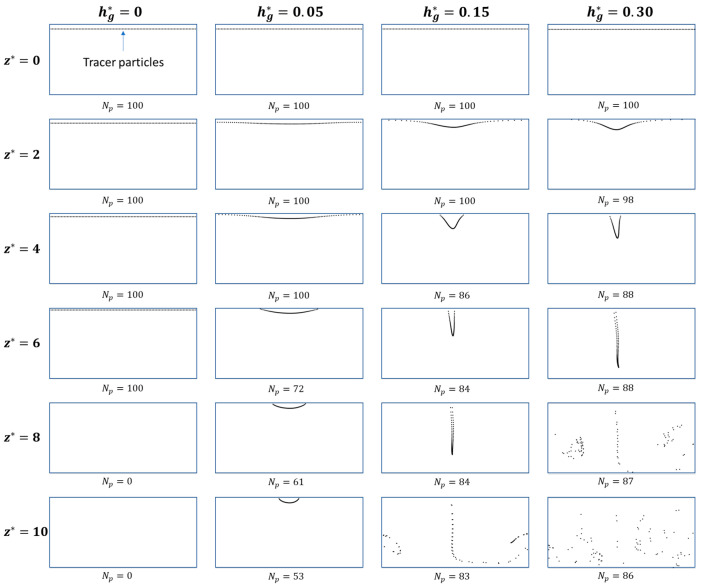
Back transport of 100 tracer particles for values of $h_{g}^{*}$ (0, 0.05, 0.15, and 0.3) when Re=100 and Rew=0.01. At the inlet ($z^{*}=0$), the particles are initially located $0.05\;h_{c}$ away from the membrane surface (see the first row). Here $N_{p}$ is the number of tracer particles found at each cross section.

In the previous section, we learned that the flow system represented by Poincaré sections is very sensitive to the groove depth when the Reynolds number is fixed. For a given groove depth, the strength of the downwelling flow is influenced by fluid inertia, but with a different dependence of $v_{dw}^{*}$ on Re at each $h_{g}^{*}$. The downwelling flows are associated with the back transport of foulants near the membrane surface. The primary factor governing the filtration performance of a membrane module is the rate of foulant transport in a feed solution near the membrane [45,46]. If a flow system is globally chaotic, the back transported foulants will be redistributed uniformly in the entire domain, which suppresses the accumulation of foulants on the membrane surface. The capability of back transport fluid particles (or foulants) within the proposed channel geometry is demonstrated in Appendix A (see Figure A1). In this regard, we attempt to characterize the foulant concentration on the membrane and its growth rate in the feed direction, with an emphasis on the effects of the groove depth and the Reynolds number on fouling mitigation.

**Figure 6:**
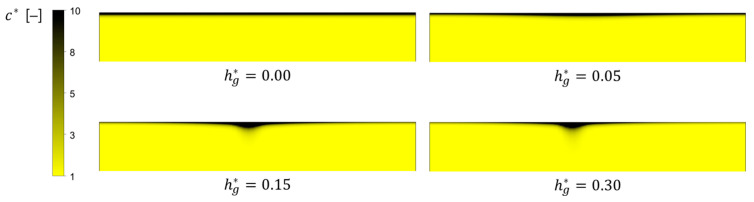
The dimensionless concentration $c^{*}\left(=c/c_{0}\right)$ near the membrane surface at a cross section located at $z^{*}=8$ for the four values of $h_{g}^{*}$, 0, 0.05, 0.15, and 0.3, when Re=100.

Both downwelling flows and chaotic advection are expected to play key roles in fouling mitigation in the flat membrane module. To demonstrate the effects of the downwelling flows on the change in the concentration polarization near the membrane surface, we plot the dimensionless concentration ($c^{*}=c/c_{0}$) distributions with $h_{g}^{*}=0$, 0.05, 0.15, and 0.3 at a cross section located at $z^{*}=8$ (Figure 6). As representative examples, the concentration distributions when Re=100 are plotted. Foulants accumulated near the membrane are dragged towards the membrane center due to two counter-rotating flows and then back transported toward the bulk region due the downwelling flows. This results in a thinner concentration boundary layer on the membrane as the groove depth increases, except for the central part of the membrane. One can observe a locally thicker layer near the center of the membrane surface, especially when $h_{g}^{*}=0.15$ and 0.3, which is caused by convective mass transport due to the two streams merging at the center, followed by downward convection. 

**Figure 7:**
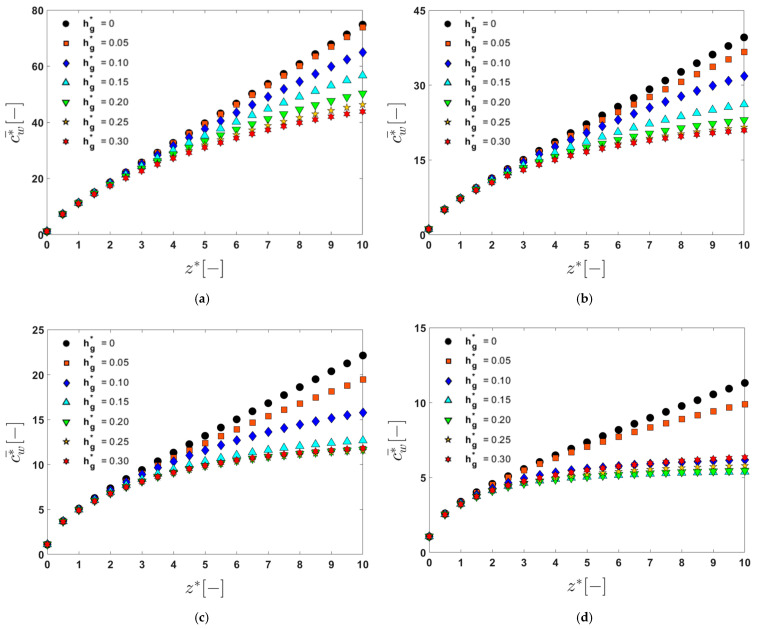
The change in the dimensionless average wall concentration (${\bar{c}}_{w}^{*}$) in the dimensionless z‒direction ($z^{*}$), affected by the dimensionless groove depth, $h_{g}^{*}$: (**a**) Re=50; (**b**) Re=100; (**c**) Re=200; (**d**) Re=500.

Since the wall concentration ($c_{w}$) varies with the lateral position at the upper edge, the line-averaged dimensionless wall concentration (${\bar{c}}_{w}^{*}$) defined by Equation (4) will be used to quantify the change in the wall concentration on the membrane surface in the z‒direction. The evolution of ${\bar{c}}_{w}^{*}$ in the feed direction is analyzed by varying Re and $h_{g}^{*}$, from which the correlation between fouling mitigation and the flow characteristics can be found. Figure 7 shows the evolution of the dimensionless concentration (${\bar{c}}_{w}^{*}$) as a function of the dimensionless axial coordinate ($z^{*})$ in the flat filtration module consisting of 10 periodic units. The dimensionless concentration (${\bar{c}}_{w}^{*}$) is a line-averaged dimensionless concentration, evaluated along the upper edge of the rectangular cross section at an axial position (see Equation (4)). The dimensionless groove depth ($h_{g}^{*}$) is varied from 0 to 0.3 with an increment of 0.05 at the four Reynolds numbers, 50, 100, 200, and 500. At each Reynolds number, an optimal groove depth minimizing ${\bar{c}}_{w}^{*}$ can be found. When Re=50 and 100, the concentration (${\bar{c}}_{w}^{*}$) decreases monotonically as the groove depth increases. Thus, as the groove depth increases, the foulant concentration decreases (within the limits of the groove depth used in this study). In the higher Reynolds number regime, where Re=200 and 500, however, a monotonic relation between ${\bar{c}}_{w}^{*}$ and $h_{g}^{*}$ no longer exists. When Re=200, the concentration is the lowest at $h_{g}^{*}=0.25$. At the higher groove depths with $h_{g}^{*}>0.2$, however, the concentration curves are almost identical. When Re=500, the optimal groove depth is found to be $h_{g}^{*}=0.15$, where ${\bar{c}}_{w}^{*}$ is minimized. The largest groove depth does not always lead to the highest level of suppression for membrane concentration development.

**Figure 8:**
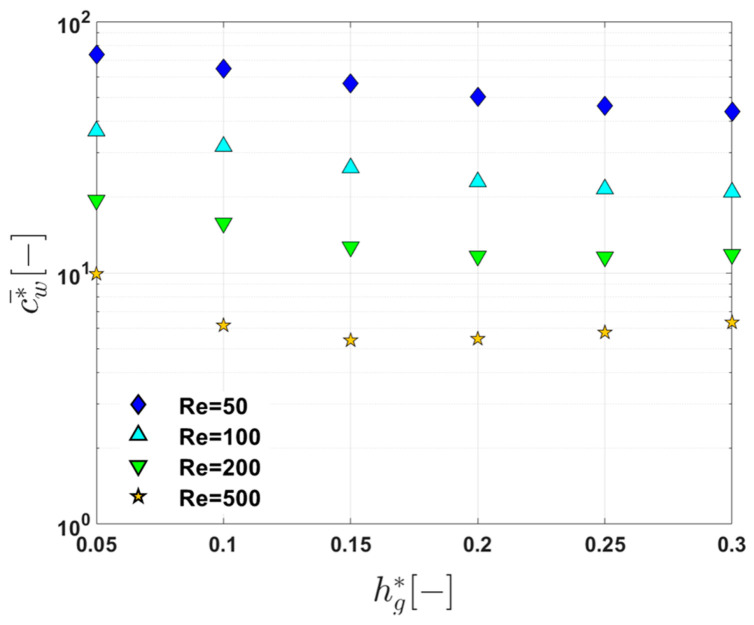
The change in the dimensionless average concentration (${\bar{c}}_{w}^{*}$) at the end of the filtration channel ($\mathrm{at}\;z^{*}=10$) as a function of the dimensionless groove depth ($h_{g}^{*}$) when Re=50, 100, 200, and 500.

To characterize the influence of the groove depth on reducing the wall concentration on the membrane surface, we plot ${\bar{c}}_{w}^{*}$ at the end of the filtration channel (at $z^{*}=10$) as a function of $h_{g}^{*}$ (Figure 8). From Figure 8, one can clearly observe the change in ${\bar{c}}_{w}^{*}$ at $z^{*}=10$ as $h_{g}^{*}$ increases at the four Reynolds numbers. The critical Reynolds number (Rec) separating the monotonic behavior and non-monotonic behavior between ${\bar{c}}_{w}^{*}$ and $z^{*}$ is approximately Rec=200. In this plot, the most interesting point is that, when Re=500, ${\bar{c}}_{w}^{*}$ at $z^{*}=10$ is minimum at $h_{g}^{*}=0.15$. Although the flow system with $h_{g}^{*}=0.15$ is not globally chaotic, the concentration curve obtained at this groove depth is the lowest (see Figure 7). Thus, the two small islands in the Poincaré section for the flow with $h_{g}^{*}=0.15$ and Re=500 (Figure 5) have a negligible effect on the concentration on the membrane surface. The lower concentration with $h_{g}^{*}=0.15$ than that with $h_{g}^{*}=0.30$ can be explained using the cross-sectional flow characteristics depicted in Figure 4. When Re=500, the downwelling flow strength for the case with $h_{g}^{*}=0.15$ is larger than that with $h_{g}^{*}=0.3$, which leads to a larger back transport lowering the concentration on the membrane. From the changes in ${\bar{c}}_{w}^{*}$ with $h_{g}^{*}$ when Re=500, it is again demonstrated that the foulant transport in a feed solution near the membrane surface is of great importance in fouling mitigation. In the case of the flat membrane filtration module, the dynamical systems of the flow (represented by the Poincaré section) and the local flow characteristics near the membrane (in this case the downwelling velocity magnitude) are closely related to the concentration on the membrane surface and subsequently to fouling mitigation. 

### 3.4. Growth Rate of the Wall Concentration

In this section, the growth rate of the wall concentration is characterized by adopting the film theory for a fully developed laminar flow in a thin rectangular channel [45,46]. In the film theory, the dimensionless concentration difference is represented by

(5) $$\frac{c_{w}-c_{p}}{c_{b}-c_{p}}=\exp\left(\frac{u_{p}\delta }{D}\right),$$

which is the relationship between the wall concentration difference and the permeate flux. In Equation (5), $c_{w}$ is the concentration on the membrane surface, $c_{b}$ the bulk concentration (which is the same as $c_{0}$ in this study), $c_{p}$ the permeate concentration (which is zero in our case since we assume that foulants are completely rejected by the membrane), and δ the concentration boundary layer (film layer) thickness. For fully developed Poiseuille flow through the thin channel, as reported in [47], the film layer thickness δ is represented by

(6) $$\frac{\delta \left(z\right)}{z}=1.475{\left(\frac{h}{z}\right)}^{\frac{2}{3}}{\left(\frac{D}{u_{max}h}\right)}^{\frac{1}{3}},$$

where $u_{max}$ is the maximum velocity in the fully developed laminar flow, h the channel half-height, and D the diffusivity. By combining Equations (5) and (6), as used in [18], one can obtain an exponential form of the increase in the dimensionless concentration, given by

(7) $${\bar{c}}_{w}^{\;*}=\exp\left(K{\left(z^{*}\right)}^{n}\right),$$

where K is a constant and n an exponent representing the growth rate of the foulant concentration in the *z*‒direction. 

To find exponent n for the data shown in Figure 6, we use a curve fitting tool in MATLAB (The MathWorks Inc., Natick, MA, USA). The fitted values of n as a function of the dimensionless groove depth ($h_{g}^{*})$ at the four Reynolds numbers, Re=50, 100, 200, and 500, are listed in Table 2.

In each row (where Re is fixed), the numbers in bold indicate the values of $h_{g}^{*}$ and n when the wall concentration growth rate is minimum at the Reynolds number. Up to the Reynolds number of 100, a deeper groove correlates with a lower exponent, showing a monotonic decrease in n with $h_{g}^{*}$. At the higher Reynolds numbers (Re=200 and 500), however, the change in n with respect to $h_{g}^{*}$ is no longer monotonic. At the two Reynolds number flows, the deepest groove does not result in the slowest growth of the wall concentration on the membrane surface, and there exists an optimal $h_{g}^{*}$ leading to the lowest n. In addition, for a given channel geometry (say $h_{g}^{*}=0.30$), the degree of fouling mitigation estimated in terms of the exponent n shows an irregular dependency on the Reynolds number. This irregularity can also be found in the mixing in laminar flows [48,49,50]. The minimum value of n (showing the lowest growth rate of the wall concentration) at a Reynolds number decreases with the increase in the Reynolds number, which indicates that increasing inertia leads to a slowdown of the growth rate of the wall concentration if a proper $h_{g}^{*}$ is chosen at the specific Reynolds number. 

In the hydrodynamic technique, utilizing chaotic advection in a flat membrane module to mitigate fouling, the groove geometry should be designed in consideration of operating conditions (in this study the Reynolds number). A chaotic flow system with a strong downwelling flow leads to the best suppression of membrane fouling, which is characterized by the wall concentration ${\bar{c}}_{w}^{*}$ and the exponent n. As observed in this study, a specific groove design showing the best performance under an operating condition does not always lead to the best performance under other operating conditions. In real applications, statistical methods such as the Taguchi method can be employed to obtain an optimal set of design variables in a filtration module [18].

### 3.5. Pressure Loss in the Constant Permeate Flux Mode 

As described in Section 2.2, the model problem used in this study falls under the operation mode with a constant permeate flux with the wall Reynolds number Rew=0.01. In a constant transmembrane pressure (TMP) mode, the increase in the resistance due to membrane fouling leads to a permeate flux decline, while in a constant permeate flux mode, TMP increases as the resistance increases [51]. Though we numerically characterize the development of the wall concentration, the concentration polarization (or fouling) is not coupled to the flow problem in the present formulation. However, the change in the pressure loss can provide an additional benchmark for comparing the performance of patterned surfaces with different groove depth. In this regard, the pressure loss in the flat membrane module that is operated in the constant permeate flux mode is investigated. We choose the dimensionless pressure loss ($\Delta p^{*}$) in a periodic unit as an indicator to demonstrate an overall influence of the patterned surface on energy consumption affected by $h_{g}^{*}$ and Re. The dimensionless pressure loss is defined by

(8) $$\Delta p^{*}=\frac{\Delta p}{\Delta p_{0}},$$

where $\Delta p_{0}$ is the pressure loss in the first periodic unit (where $0\leq z^{*}\leq 1$) without grooves (i.e., $h_{g}^{*}=0$) at a specific Re, and Δp is the pressure loss in the periodic unit with a groove depth $h_{g}^{*}$ at the corresponding Re. 

**Figure 9:**
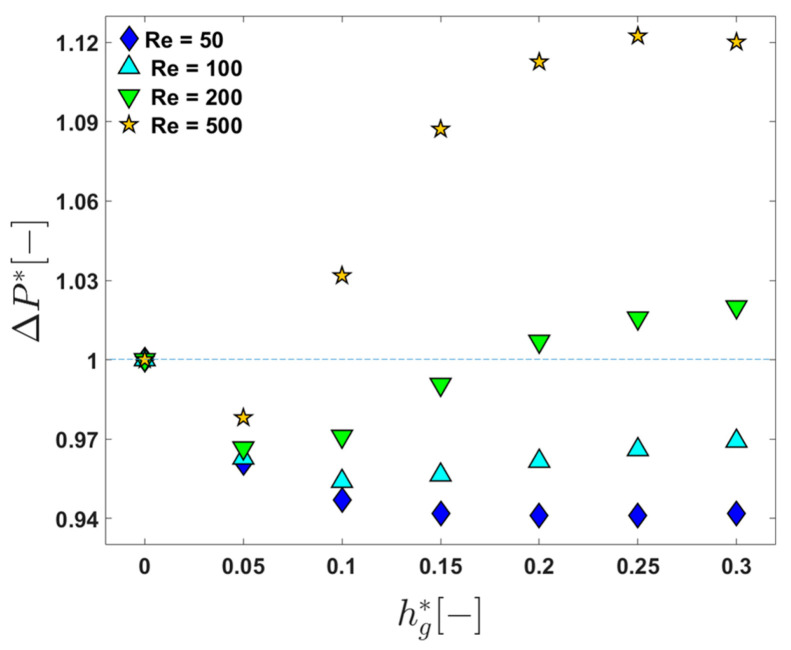
The change in the dimensionless pressure loss $\Delta p^{*}$ in one periodic unit as a function of the dimensionless groove depth ($h_{g}^{*}$) when Re=50, 100, 200, and 500.

Figure 9 shows the change in $\Delta p^{*}$ in one periodic unit as a function of $h_{g}^{*}$ at the four Reynolds numbers, Re=50, 100, 200, and 500. When Re=50 and 100, the pressure loss $\Delta p^{*}$ in a channel with grooves is always less than 1, demonstrating a positive influence of the patterned surface in terms of energy efficiency at this Reynolds number. The lower pressure losses compared to that in the channel without grooves are thought to be caused by the effective slip [52] due to the flow in the herringbone-shaped grooves, lowering viscous dissipation. In addition, the positive influence of the patterned surface in terms of energy consumption, lowering the pressure loss, is dominant in the flow regime with Re≤100 regardless of the groove depth. When Re=200, $\Delta p^{*}$ is larger than 1 at the deeper grooves ($h_{g}^{*}\geq 0.2)$, but the increase in the pressure loss is less than 2%. At the highest Reynolds numbers (Re=500), $\Delta p^{*}$ is larger than 1 except for the case with $h_{g}^{*}=0.05$. From the viewpoint of pressure loss in the constant permeate flux mode, the flow at Re=500 is not as efficient as that at the lower Reynolds numbers, but it should be noted that the wall concentration and the growth rate are the lowest at Re=500 when a proper groove depth is chosen (see Figure 8 and Table 2). The reduction in the wall concentration leads to the decrease in the resistance from the adsorption of foulant compared to that of the flat plain channel with $h_{g}^{*}=0$, but at the cost of higher pressure losses. The implementation of a sophisticated boundary condition, considering the effects of the TMP and osmotic pressure on the permeate flux, will be a subject for future study on the flat membrane module utilizing chaotic advection. 

## 4. Conclusions

We carried out a numerical investigation on the flow and mass transfer in a flat membrane module incorporating a herringbone-shaped surface used to induce chaotic advection in the filtration module. A computational domain was chosen using the symmetry and periodicity of the surface pattern. The groove depth was chosen as the only geometrical variable with the dimensionless groove depth $h_{g}^{*}$ ranging from 0.05 to 0.3. The Reynolds number varied from 50 to 500. Assuming a constant permeate flux, the wall Reynolds number (Rew) was fixed to 0.01. The flow characteristics in the filtration channel were revealed to be sensitive to the groove depth and fluid inertia, which was demonstrated by the Poincaré sections and cross-sectional velocity vectors. At the lower groove depth ($h_{g}^{*}=0.05$), the flow system was regular (not chaotic). As the groove depth increased, the flow system became partially or globally chaotic, which could represent a promising means to mitigate membrane fouling. In addition, the downwelling flow magnitudes were compared by varying the groove depth since the downwelling flow was a key factor inducing the back transport of foulants in the flat membrane module. Under the lower Reynolds number flow (at Re=100), a deeper groove depth correlated with a stronger downwelling flow. When Re=500, however, the deepest groove (with $h_{g}^{*}=0.30$) did not give rise to the strongest downwelling flow, but a moderate groove depth ($h_{g}^{*}=0.15$) led to the strongest downwelling flow. For the quantitative analysis of the fouling mitigation, a dimensionless line-averaged wall concentration (${\bar{c}}_{w}^{*}$) on the membrane surface was defined, and its evolution in the feed direction was evaluated. We were able to choose a proper groove depth at a specific Reynolds number, minimizing ${\bar{c}}_{w}^{*}$ and the exponent n (representing the growth rate of ${\bar{c}}_{w}^{*}$). A groove geometry showing the lowest fouling (in terms of the wall concentration) at a specific Reynolds number does not always result in the best performance in the other flow regime. Finally, the pressure loss in the filtration module was characterized to evaluate the influence of $h_{g}^{*}$ and Re on energy consumption. The present numerical study demonstrated the feasibility of the use of chaotic advection induced by the patterned surface to mitigate fouling in the flat membrane module. 

## Figures and Tables

**Table 1:** The number of elements and the minimum element size of the meshes used to check convergence with mesh refinement. Here, $h_{c}$ is the channel height.

Mesh	Number of Elements	Minimum Element Size
M1	8,608,896	0.01$\;h_{c}$
M2	16,685,136	0.005$\;h_{c}$
M3	30,380,832	0.0025$\;h_{c}$
M4	41,685,120	0.00125$\;h_{c}$

**Table 2:** The fitted values of n at the three dimensionless groove depths when Re=50, 100, 200, and 500. In each row, numbers in bold indicate the values of $h_{g}^{*}$ and n when the growth rate is minimum at a specific Reynolds number.

Re	$h_{g}^{*}$	n
50	0.05	0.2347
	0.10	0.2163
	0.15	0.2005
	0.20	0.1866
	0.25	0.1773
	**0.30**	**0.1712**
100	0.05	0.2419
	0.10	0.2191
	0.15	0.1916
	0.20	0.1749
	0.25	0.1668
	**0.30**	**0.1633**
200	0.05	0.2524
	0.10	0.2084
	0.15	0.1745
	0.20	0.1627
	**0.25**	**0.1625**
	0.30	0.1666
500	0.05	0.2580
	0.10	0.1577
	**0.15**	**0.1342**
	0.20	0.1411
	0.25	0.1559
	0.30	0.1771

## Data Availability

Not applicable.

## List of Symbols

c	Concentration of foulant, mol/m^3^
$c_{b}$	Concentration in the bulk, mol/m^3^
$c_{0}$	Concentration at the inlet, mol/m^3^
$c_{p}$	Concentration in the permeate, mol/m^3^
$c_{w}$	Concentration on the wall (membrane surface), mol/m^3^
$c_{w}^{*}$	Dimensionless wall concentration
${\bar{c}}_{w}^{*}$	Dimensionless line-averaged wall concentration
D	Diffusivity of foulants, m^2^/s
δ	Thickness of the concentration boundary layer (film layer), m
h	Half-height of a thin slit channel, m
$h_{c}$	Channel height, m
$h_{g}$	Groove depth, m
$h_{g}^{*}$	Dimensionless groove depth
$l_{p}$	Length of a periodic unit of the channel, m
n	Unit outward normal vector
*n*	Exponent of the growth rate of the wall concentration
p	Pressure, Pa
Pe	Péclet number
Re	Reynolds number
Rec	Critical Reynolds number
Rew	Wall Reynolds number
u	Velocity vector, m/s
$\bar{u}$	Uniform normal velocity at the inlet, m/s
$u_{max}$	Maximum velocity in the fully developed laminar flow through a thin slit, m/s
$u_{p}$	Permeate velocity, m/s
$v_{dw}^{*}$	Dimensionless downwelling velocity magnitude
$w_{c}$	Channel width, m
$w_{g}$	Groove width, m
$z^{*}$	Dimensionless z–coordinate
**Greek Letters**	
$\Gamma_{i}$	Inlet boundary
$\Gamma_{o}$	Outlet boundary
$\Gamma_{s}$	Non-permeable solid boundary
$\Gamma_{w}$	Membrane surface
δ	Concentration boundary layer thickness, m
μ	Viscosity, Pa∙s
θ	Groove angle, °

## Appendix A. Back Transport of Tracer Particles

To demonstrate back transport of foulants due to the two counter-rotating flows, 100 tracer particles are introduced at the inlet, and they are tracked to the end of the channel (Figure A1). In this case, the Reynolds number of the bulk flow and the wall Reynolds number are Re=100 and Rew=0.01, respectively. We use channels with four different groove depths, $h_{g}^{*}=0$, 0.05, 0.15, and 0.3, to see the influence of $h_{g}^{*}$ on the back transport capability under the specific operating conditions. As depicted in the first row of Figure A1, the particles initially are located at $0.05\;h_{c}$ away from the membrane surface (the upper edge of the rectangular cross section). When $h_{g}^{*}=0$ (i.e., a flat plain channel without any back transport mechanism), the tracer particles are drawn to the membrane surface by the permeation drag, crossing the upper membrane surface after $z^{*}=6$. As $h_{g}^{*}$ increases, however, the number of particles ($N_{p}$) found at the outlet increases with $h_{g}^{*}$. At the deepest groove depth ($h_{g}^{*}=0.3$), 86% of the particles introduced through the inlet remain at the outlet without going out of the computational domain following the permeate flow.
